# Reactive Ion Etching in the Gaseous Electronics Conference RF Reference Cell

**DOI:** 10.6028/jres.100.033

**Published:** 1995

**Authors:** M. L. Brake, J. T. P. Pender, M. J. Buie, A. Ricci, J. Soniker, P. D. Pochan, P. A. Miller

**Affiliations:** Department of Nuclear Engineering, University of Michigan, Ann Arbor, MI 48109; Sandia National Laboratories, Albuquerque, NM 87106

**Keywords:** discharge, etch depth, etch rate, etching, gaseous electronics, radio frequency, reactive ion etching

## Abstract

This paper describes the results of using the GEC reference cell as a reactive ion etcher. Silicon wafers with layers of polysilicon and silicon dioxide on crystaline silicon patterned with photoresist have been investigated with fluorine and chlorine chemistries. Scanning electron microscopy (SEM), profilometry, and refraction techniques were used to determine the etch parameters such as etch rate, uniformity and selectivity. The discharges are in general monitored by measuring the optical emission spectroscopy and the bias voltages. For fluorine chemistries, etch rates ranged from 5 nm/min to 177 nm/min, and for chlorine chemistries, etch rates ranged from 25 nm/min to 90 nm/min. Depending upon the discharge and chemistry conditions, similar etch rates and etch patterns of different GEC cells were obtained. Etch rates and relative fluorine concentrations obtained from a commercial etcher were compared to the GEC reference cell and were found to be similar although the GEC cell generally gave lower etch rates than the commercial etcher.

## 1. Introduction

The increased use of radio-frequency (rf) glow discharges in microelectronics fabrication has resulted in the need for greater comprehension and control of the discharges. One of the most important factors affecting plasma behavior is the geometry of the discharge chamber, as minor alterations in design result in large variations in plasma parameters and hence in etching results. In industry, apparently identical commercial etchers can behave differently. Because of the number of different systems used, it is difficult to compare data from machines of differing properties. A standardized discharge chamber was designed so that models and measurements at different locations could be directly compared. This chamber is known as the GEC Reference cell. The cell was designed as a standardized chamber with the ultimate goal of understanding the processes that take place in discharges used in microelectronics fabrication. This paper describes the etching that has been performed in several GEC cells, and also gives the results of one study which compared the etching performance of the GEC cell with a commercial etcher. A detailed description of the GEC cell can be found in Ref. [1].

In spite of the fact that the GEC cell was designed as a test bed for diagnosing 13.56 MHz parallel plate discharges similar to those used in industry, only a few groups have examined etching in the GEC cell [2–10]. All of the groups have looked at the etching of silicon, polysilicon and silicon dioxide, but none have tried to make an actual device. All of the groups have used the GEC cell in the reactive ion etcher mode (RIE), where the bottom electrode is powered and the upper electrode and walls of the chamber are grounded. To date there has been only one study comparing the etching ability of the GEC cell to a commercial reactor [2, 3] although a commercial cell and a GEC cell were compared in studies of particle formation [10]. One major disadvantage of the current GEC cell is the 10 cm electrodes were designed when most groups were using 7.6 cm wafers. Many labs have since upgraded to 10 cm and larger wafers.

Different chemistries have been examined by different groups. Pender and Buie [2–5] have studied plasma and wafer uniformity as well as etch performance by concentrating on CF_4_ and CF_4_/O_2_ to etch photoresist patterned wafers with and without silicon dioxide films covered with polysilicon layers. Lane [6] has examined end-point detection of polysilicon, oxide, and nitride films using SF_6_/O_2_, CF_4_/H_2_, and CF_4_/O_2_, respectively. Pochan and Miller [7] have investigated GEC etch performance by etching silicon covered with polysilicon, patterned with photoresist using Cl_2_ and Cl_2_/HBr chemistries. Oh et al. [ 8] have etched silicon and silicon dioxide in CF_4_/CHF_3_ mixtures while developing a diode laser absorption technique. Splichal and Anderson [9] have studied resist patterned wafers over a thermal oxide layer while testing optical emission diagnostics for process systems in CF_4_/CHF_3_. Anderson et al. [10] have etched oxide wafers in both a Drytek Quad Model^3^ 480 RIE and in the GEC cell in C_2_F_6_-CHF_3_, but studied particle contamination rather than etch performance [10]. Look for an accompanying article in this journal for details of this experiment. A summary of the above mentioned studies is given below.

## 2. Experimental Technique

**Fig. 1:**
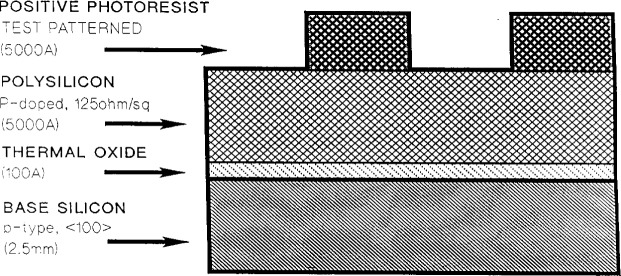
Schematic of a typical wafer.

As mentioned in the introduction, all of the experiments described in this paper used the GEC cell in the reactive ion etcher mode; the top electrode and side walls of the chamber were grounded and the bottom electrode was powered with 13.56 MHz rf power. Usually a matching network was used and in some cases a shunt circuit was also used. The electrode spacing was typically 2.54 cm. At the Universities of Michigan [2–5] and New Mexico [8–9] the wafers were placed on the bottom electrode through a hinged optical port that opened up to the atmosphere to allow access to the cell. Sandia National Laboratories [7] and the Rochester Institute of Technology [6] used a load locked system to place the wafer on the bottom electrode under vacuum conditions. The wafers etched in these studies had a typical pattern shown in Fig. 1. A typical wafer consisted of either p- or n-type silicon covered by a thin thermal oxide layer, followed by a polysilicon layer and topped off with a layer of patterned photoresist. In some of the uniformity studies by Buie [4, 5] photoresist patterned silicon wafers without the poly and oxide layers were used since etch depth uniformity was the outcome of interest. Note that the photoresist is used to mask off part of the wafer for etch depth determination. The masks in general are simply test patterns rather than patterns used in making electronic devices.

Etch depth was determined by measuring the etch thickness before and after the etch (etch times are typically 30 min for fluorine based chemistries and a few minutes for the chlorine based chemistries) using a reflectometer [2,3,7]. Scanning Electron Microscopy (SEM) [2,3,7] and profilometry [4, 5] were used to examine the etch depth (typically of trenches) after the etches to examine both the microscopic and macroscopic features. Since etching plasmas use very reactive gases, there are not many suitable *in situ* diagnostics. The least invasive and the most common *in situ*, nonperturbing diagnostic used in the etch experiments was optical emission spectroscopy (OES). OES was used to identify the constituents of the plasma [2,4–6,9] and to determine the endpoint of the etch using actinometry [2,3,6], the ratio of a fluorine emission line to an argon emission line (i.e., determined when the etch had removed the polysilicon layer and had reached the oxide layer), and spatially resolved optical emission of one wavelength to examine the uniformity of the plasma [4, 5]. Diode laser absorption measurements were also used to determine various chemical constituents of the plasma [8]. In most cases the bias voltage was also measured during the etch. Residual gas analyzers have also been used to examine the gas species during etching [6]. A summary of the results is given in Table 1.

## 3. Summary of Etching Experiments

**Fig. 2:**
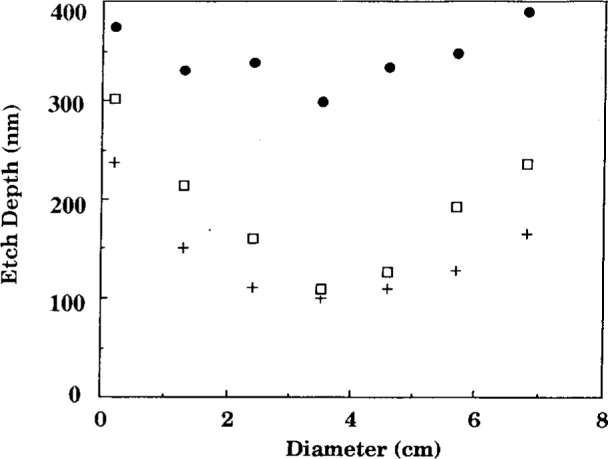
Etch depth as a function of diameter across the wafer for a 15 W CF_4_/Ar discharge. Note that etch uniformity improves as the pressure is decreased: (•) 10 Pa (75 mT), (□) 20 Pa (150 mT), and (+) 33 Pa (250 mT), (see Ref. [4]).

**Fig. 3:**
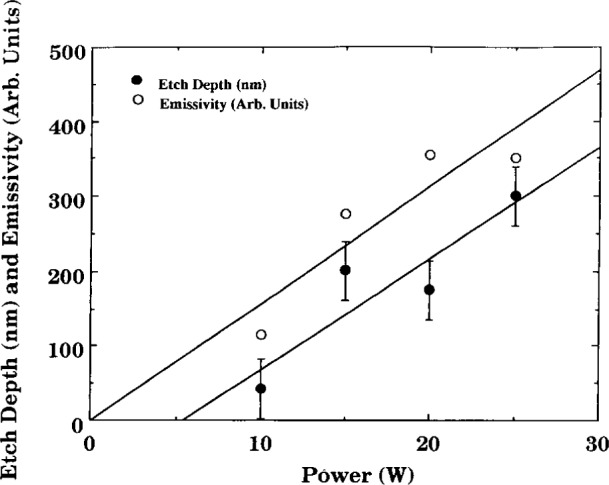
Etch Depth at the center of the wafer (•) and Emissivity (○) and as a function of power for a pressure of 26.6 Pa (200 mTorr) in a CF_4_/Ar discharge. Note that both scale linearly (within error) with power.

**Fig. 4:**
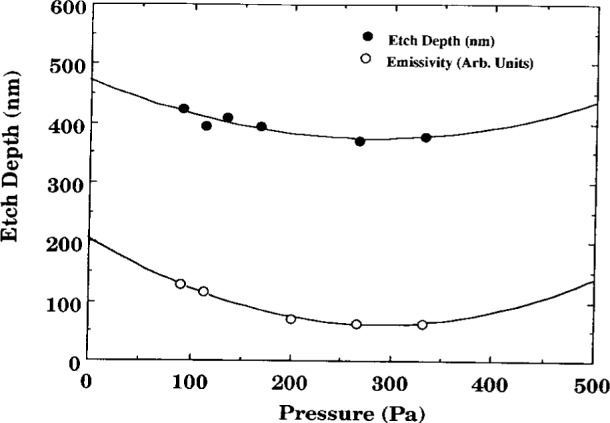
Etch Depth (•) and emissivity (○) as a function of pressure while etching with CF_4_/Ar/O_2_ discharges at 20 W with 4 % oxygen added to the plasma to prevent polymerization. Note that both emissivity and etch depth scale quadratically with pressure, (see Ref. [4]).

Buie et al. [4, 5] have investigated the correlation between the spatially resolved optical emission spectroscopy (SR-OES) and the uniformity of the etch in CF_4_ and CF_4_/O_2_ discharges. SR-OES was taken during the etch of photoresist patterned silicon wafers (i.e., without a layer of polysilicon or silicon oxide). The first major result of this study was to show that the plasma etched nonuniformly, which matched previous optical emission studies of Pender et al. [13] and Djurovic et al. [14]. That is, the discharge etched the edges much more quickly than the middle. See Fig. 2 for typical results. Note that lower pressure discharges result in more uniform macroscopic etching. The second result of this study [4, 5] was to show that there is a correlation between the emissivity (calculated from Abel inversion of the collected parallel rays of light from the discharge) and the etch depth as a function of power and pressure. A physical model for this correlation, illustrated in Figs. 3 and 4, has not been developed at this time. Note that 4 % oxygen had to be added to reduce polymerization. In general etch rates of 10 nm/min to 15 nm/min were measured for 15 W to 20 W and a pressure range of 6.7 Pa to 33.3 Pa (50 mTorr to 250 mTorr). Fluorine loading effects were also investigated in this study. Three sets of experiments were conducted. The etch depths resulting from a 7.6 cm (3 in) wafer sitting on the bare aluminum electrode, a 7.6 cm wafer sitting on a 10 cm (4 in) wafer on the electrode, and a 7.6 cm wafer sitting on a quartz plate sitting on the aluminum electrode were measured. The results of this study showed all three conditions gave similar nonuniform etch profiles and etch depths within experimental error. Thus, fluorine loading is not the cause of nonuniform etches.

In a second set of experiments by Pender [2, 3], actinometry was used to study etch rates in the cell itself and used to compare with etch studies in a commercial cell. For results of these studies see Secs. 4 and 5. These experiments were conducted in CF_4_ at flows of 14.9 μmol/s (20 sccm) with power densities ranging from 25 mW/cm^3^ to 60 mW/cm^3^. The etch rates ranged from 5 nm/min to 20 nm/min for 15 min etches.

Pochan and Miller etched in Cl_2_ and Cl_2_/HBr plasmas in a Sandia GEC cell [7]. Their wafers were 10 cm (4 in) diameter of p-type silicon covered with 12 nm of oxide, 450 nm of polysilicon and 500 nm patterned photoresist. They observed etch rates of 50 nm/min to 90 nm/min with flows of 14.9 μmol/s (20 sccm) of Cl_2_ and 13.3. Pa (100 mTorr) and peak to peak voltage (V_p–p_) of 200 V, RF (10 W) power. The Cl_2_/HBr rates span 25 nm/min to 50 nm/min for flows of 11.2 μmol/s (15 sccm) of Cl_2_, with flows of 3.7 μmol/s (5 sccm) of HBr, at 13.3 Pa (100 mTorr) and of V_p–p_ = 200 V. Scanning electron microscope profiles show that the addition of HBr changes the etch profile from isotropic to anisotropic. The etch rate as a function of power and the etch uniformity will be discussed in Sec. 4.

Oh et al. [8] has been developing a diode laser absorption diagnostic to be used for *in situ* measurements of CF_4_, CF_2_, and CF_2_O, as well as for end point detection during the etching of SiO_2_. Two types of wafers were used in their study, a blanket thermal oxide (800 nm–1000 nm) on n-type silicon wafers for the end-point determination and a half-and-half oxide/polysilicon (800 nm–1000 nm) layered p-type wafers for selectivity determination. The wafers used in the selectivity studies were also patterned with a 500 nm layer of photoresist. The film thickness for the selectivity experiments was measured before and after etching with an interferometer. The blanket wafers were etched to end point. The oxide etch rates ranged from 9.28 nm/min to 176.74 nm/min using pressures ranging from 40 Pa to 93 Pa (300 mTorr to 700 mTorr), various mixtures of CF_4_/CHF_3_ flow rates, and peak to peak voltages of 500 V to 900 V. The CHF_3_ ranged from 4.8 % to 33.3 %. The poly/Si etch rates ranged from 1.3 nm/min to 115 nm/min over the same pressure and applied voltage range as the oxide studies. Oxide to poly selectivity was found to range from 0.93 to 3.05. The conclusions of their study were that by monitoring the feed gas dissociation it might be possible to provide an indicator which could be related to process parameters such as etch rate, selectivity, and anisotropy. They did not draw any conclusions regarding the etching ability of the GEC cell since their main objective was to study this new diode laser diagnostic.

Splichal and Anderson [9] have examined the application of spectral signature analysis techniques to silicon dioxide etching in CF_4_/CHF_3_. They used the GEC cell to etch 10 cm wafers with 830 nm of thermal oxide covered with a 1.7 μm positive resist pattern that exposed approximately 60 % of the oxide film. They measured etch rates ranging from 8.5 nm/min to 59.8 nm/min for applied peak to peak voltages of 500 V to 800 V, pressures of 40 Pa to 80 Pa (300 mTorr to 600 mTorr) and CHF_3_ of 3.2 % to 41.2 %. They concluded that the application of chemometrics to OES was a promising technique for plasma monitoring.

At the Rochester Institute of Technology, Lane and Grimsley [12] are investigating deep etching (3 μm to 5 μm) of crystalline silicon with the goal of achieving very smooth etched surfaces, both horizontal and sidewall. In this work, the primary etchant is SF_6_ with small additions of various other gases. They are finding that very small quantities (2 % to 5 %) of oxygen, hydrogen and other gases have a large effect on the smoothness of the etched surfaces.

## 4. A Comparison of Data Taken on Different Cells Using the Same Technique

**Fig. 5:**
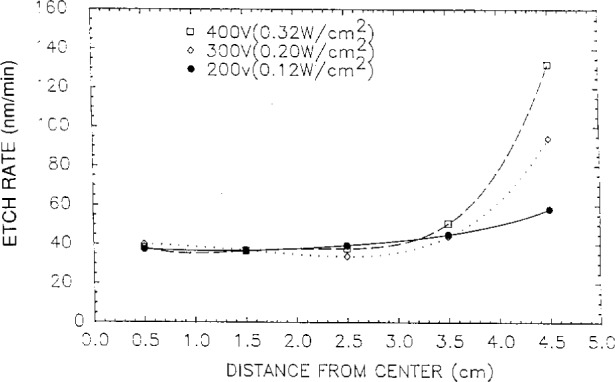
Etch rate measured from the center of the wafer for three applied voltages in a Cl_2_ etch at 13.3 Pa (100 mTorr).

**Fig. 6:**
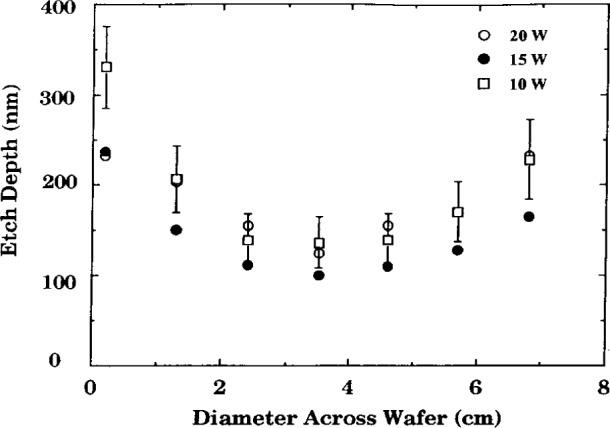
Etch depth at the center of the wafer as a function of diameter across the wafer for three different powers (20 W (○), 15 W (•), 10 W (□)) where the wafers were etched in CF_4_/Ar for 30 minutes at 33 Pa (250 mTorr).

**Fig. 7:**
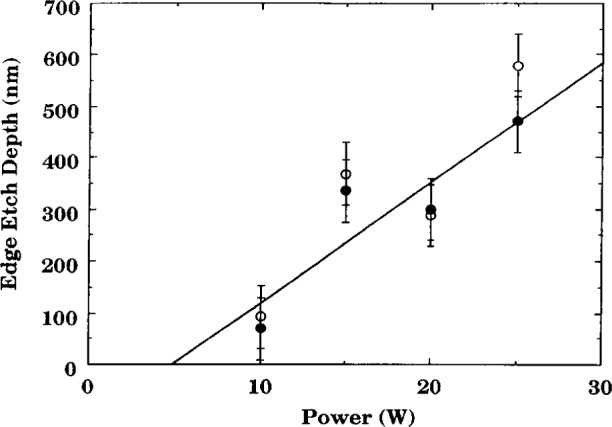
Etch depth at the two edges of the wafer as a function of power for wafers etched in CF_4_ for 30 min at 36.6 Pa (200 mTorr).

**Fig. 8:**
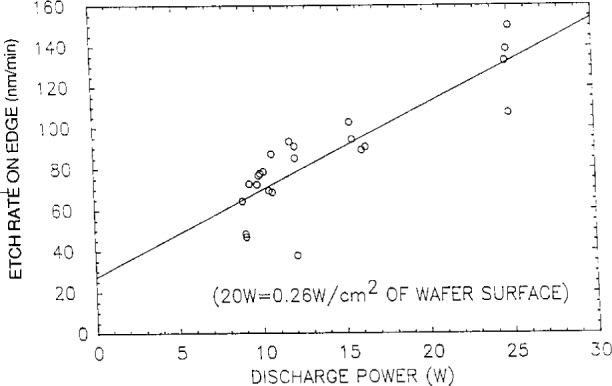
Etch rates at the edge of the wafer as a function of power for wafers etch in Cl_2_ at 13.3 Pa (100 mTorr).

**Fig. 9:**
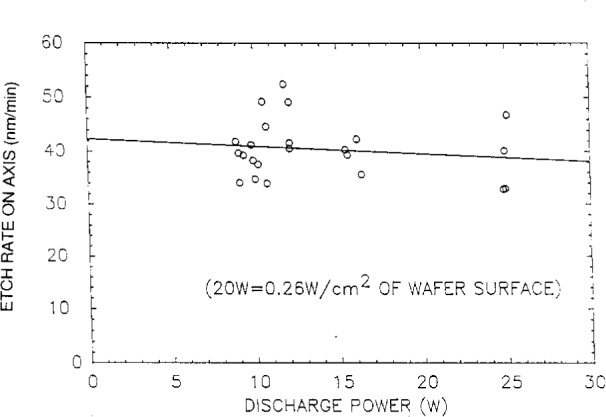
Etch rate at the center of the wafer as a function of power for wafers etched in Cl_2_ at 13.3 Pa (100 mTorr).

Even though etch chemistries were different, the etch profiles (etch rate or depth versus distance across the wafer) for wafers illustrated in Fig. 1 were very similar for etching experiments performed at the Univ. of Michigan [2–5] and Sandia [7]. They showed large etch depths at the edge of the wafers compared to the center. Michigan generally etched between 15 min and 30 min in CF_4_ sometimes with O_2_ added, whereas Sandia etched for a few minutes using Cl_2_ occasionally adding HBr. Both systems showed an increase in nonuniformity at the edges with increasing applied voltage or power, see Figs. 5 and 6. The etch rate at the edges showed large increases with power, see Figs. 7 and 8. There is however a disagreement in the functional dependence of rate with increasing power at the center of the wafer. The Michigan [5] results show an increase in etch depth at the center (see Fig. 3) as a function of power. The Sandia [7] group showed no increase in etch rate in the center of the wafer as a function of power, see Fig. 9. This may be due to chemistry effects but this result needs to be examined further. Also, Sandia etched through polysilicon to oxide whereas the Michigan group etched directly into the Si wafer.

## 5. A Comparison with a Commercial RIE

A SEMI Group 1000 TP/CC RIE, housed in a clean room, was chosen for comparison with the GEC cell [2, 3]. Both are parallel plate, rf systems operating in an RIE mode. Both introduced gas into the discharge via a showerhead configuration in the upper electrode. See Table 2 for a comparison of the two systems. Note from the table that the major difference between the two systems was the size and spacing of the electrodes. The etch depth was determined by measuring the poly thickness before and after the etch using a reflectometer and the fluorine concentration was monitored in situ with actinometry, (a process where the intensity of a fluorine optically emitted spectral line is compared to an argon emitted line). Since the distance between electrodes on this GEC cell was fixed, the effect of changing the plate spacing on the GEC cell was not examined. (The SEMI-Group RIE has movable electrodes.)

**Fig. 10:**
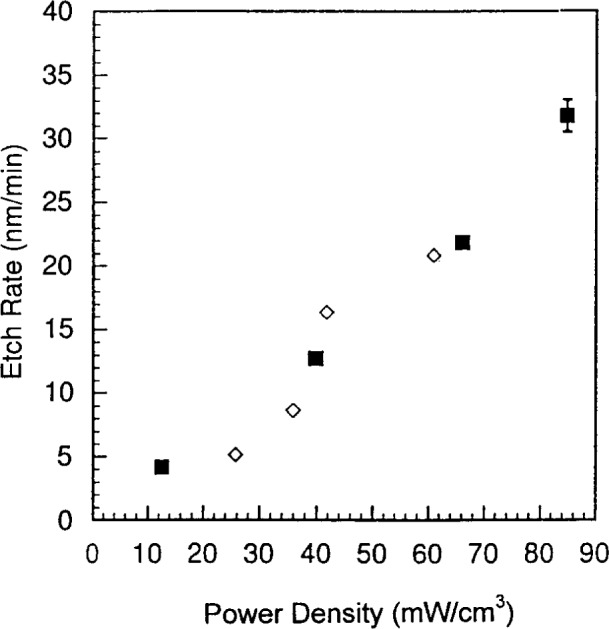
Etch rate as a function of absorbed power at 10 Pa (75 mTorr) for the GEC cell (◊) and the SEMI Group RIE (■) for wafers etched in CF_4_/Ar for 30 min.

Silicon wafers with 400 nm polysilicon over 200 nm thermally grown oxide (SiO_2_), (unpatterned) were etched in CF_4_ with 4% argon added for actinometry. The etches were performed for pressures ranging from 10 Pa–20 Pa (75 mTorr to 150 mTorr) at a flow of 22.4 μmol/s (30 sccm). See Figs. 10 through 12 for typical results. Note that the etch depths were measured at the center of the wafer for wafers etched in both machines. The final results of this study are that the two cells give similar etch rates and fluorine concentrations if the plate spacing, the flow rate and the power density are similar and, when possible, the bias voltage is the same.

In this same study, the power absorbed by the cells was determined with simple circuit models [2]. The circuit model used for the GEC was that developed by Miller and Kamon [11], and for details of the model used for measuring and calculating the power of the commercial cell, see Ref. [2]. As seen in Figs. 13a and 13b, the power measured from the power supply was actually much larger than the power absorbed by the discharge for both the GEC and SEMI-Group cells.

## 6. Conclusions

The GEC Reference Cell has been used as an etcher of silicon materials at four locations, University of Michigan, University of New Mexico, Rochester Institute of Technology, and Sandia National Laboratories. In general, similar current and voltage waveforms gave similar etch rates and etch patterns in all four cells depending upon the chemistry. The resulting etch of the wafers however was not uniform. This nonuniformity can be examined with optical emission spectroscopy and other diagnostics (see accompanying papers in this Special Issue of this journal). In one study, the GEC as used as a reactive ion etcher was compared to a commercial parallel plate system, and was found to give lower etch rates but similar etch patterns.

## Figures and Tables

**Fig. 11:**
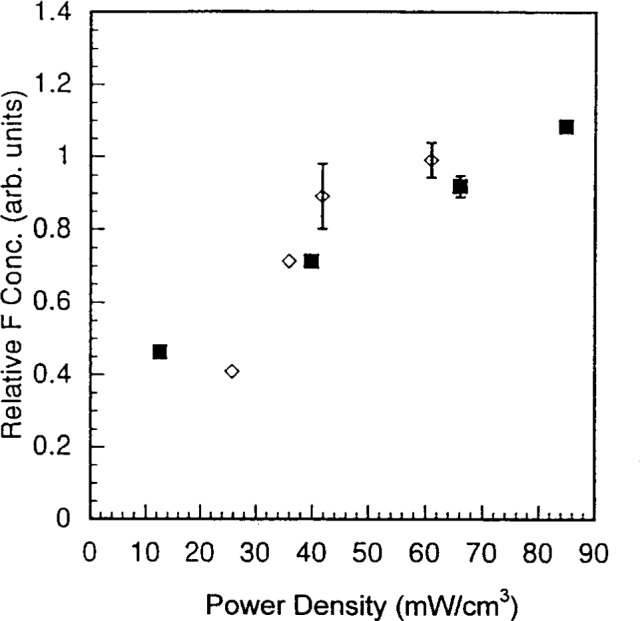
Fluorine concentration as a function of power density at 10 Pa (75 mTorr) for the GEC cell (◊) and the SEMI Group RIE (■) for wafers etched in CF_4_/Ar for 30 min.

**Fig. 12:**
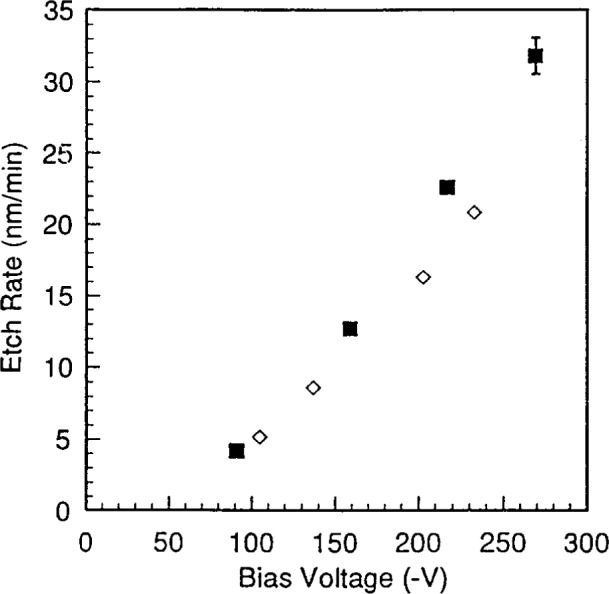
Etch rate as a function of bias voltage at 10 Pa (75 mTorr) for the GEC cell (◊) and the SEMI group RIE (■) for wafers etched in CF_4_/Ar for 30 min.

**Fig. 13a:**
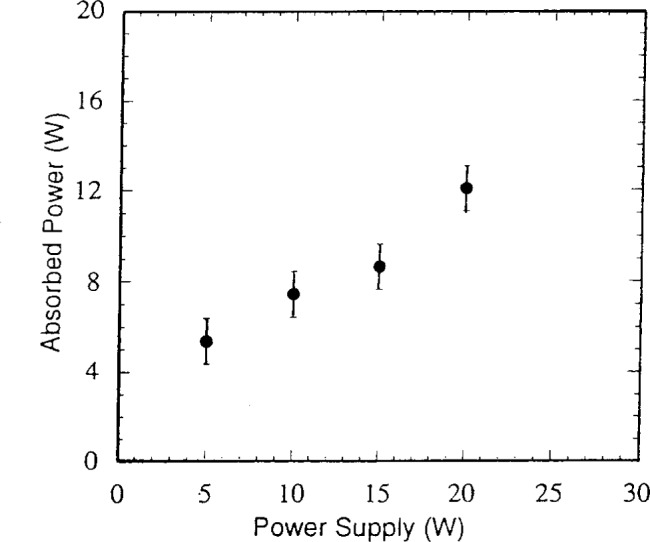
Absorbed power as a function of power read from the power supply dial on the ENI power supply used with the GEC Reference Cell.

**Fig. 13b:**
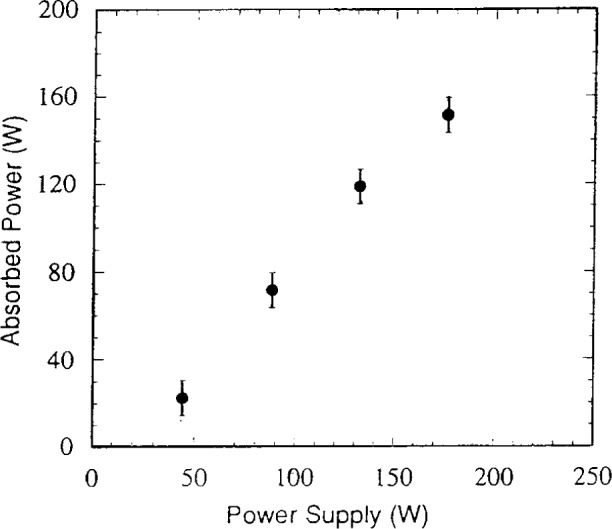
Absorbed power as a function of power read from the power supply dial on the SEMI Group RIE.

**Table 1:** A comparison of GEC reference cell etching results

Etch rate (nm/min)	Pressure (Pa)	Applied voltage (V)	Chemistry	Material	Ref.
10 to 15	6.7 to 33.3	190 to 300 V	Fl	Si	[4,5]
5 to 20	6.7 to 33.3	80 to 300V	Fl	poly	[2,3]
50 to 90	13.3	200 V	Cl	poly	[7]
25 to 50	13.3	200 V	Cl, Br	poly	[7]
9 to 177	40 to 93	500 to 900 V	Fl	oxide	[8]
1.3 to 115	4 to 93	500 to 900 V	Fl	poly	[8]
8.5 to 60	40 to 80	500 to 900 V	Fl	oxide	[9]

**Table 2:** A comparison of the construction of the SEMI Group RIE and GEC Reference Cell

	SEMI Group	UM GEC Ref. Cell
Electrode material	Aluminum	Aluminum
Powered electrode	Lower	Lower
Insulator	Ceramic	Teflon
Upper electrode dia.	40 cm	10 cm
Lower electrode dia.	30 cm	10 cm
Electrode spacing	2.5 cm to 15 cm	2.5 cm
Chamber diameter	43 cm	25 cm
Frequency	13.56 MHz	13.56 MHz
Power Supply	RFX600 600 W	ENI AGC-5 500 W
Matching network	MCS ATN 500B	ENI
Pressure	6.7 Pa–133 Pa (50 mTorr to 1 Torr)	6.7 Pa–133 Pa
CF_4_ flow rate	0 sccm–40 sccm	0 sccm–40 sccm

## Footnotes

3 Certain commercial equipment, instruments, or materials are identified in this paper to foster understanding. Such identification does not imply recommendation or endorsement by the National Institute of Standards and Technology, nor does it imply that the materials or equipment identified are necessarily the best available for the purpose.
